# The occurrence of invasive cancers following a diagnosis of breast carcinoma *in situ*

**DOI:** 10.1038/sj.bjc.6604524

**Published:** 2008-07-29

**Authors:** D Robinson, L Holmberg, H Møller

**Affiliations:** 1 Thames Cancer Registry, Division of Cancer Studies, King's College London, 1st Floor Capital House, 42 Weston Street, London SE1 3QD, UK; 2 Cancer Epidemiology Section, Division of Cancer Studies, School of Medicine, King's College London, 3rd Floor Bermondsey Wing, Guy's Hospital, London SE1 9RT, UK

**Keywords:** breast carcinoma *in situ*, second cancers, hormonal therapy, uterine cancer

## Abstract

Approximately 1 in every 600 women attending breast-screening programmes in the United Kingdom is diagnosed with breast carcinoma *in situ* (BCIS). However, there is little information on the occurrence of subsequent cancers (other than second breast cancers) in these women. We investigated the occurrence of invasive cancers in 12 836 women diagnosed with BCIS in southeast England between 1971 and 2003, using data from the Thames Cancer Registry. A greater than expected number of subsequent cancers was found for two sites: breast (standardised incidence ratio (SIR) 1.96; 95% confidence interval (CI) 1.79–2.14) and corpus uteri (SIR 1.42; 95% CI 1.11–1.78). For subsequent ipsilateral breast cancer in those treated with breast conservation, the excess was independent of the time since diagnosis of BCIS, whereas for subsequent contralateral breast cancer, there was a steady decline in excess over time. For subsequent uterine cancer, the excess became statistically significant only at >5 years after BCIS diagnosis, consistent with a treatment effect. This was further supported by Cox regression anaysis: the risk of subsequent uterine cancer was significantly increased in women receiving hormonal therapy compared with those not receiving it, with a hazard ratio of 2.97 (95% CI 1.84–4.80).

Currently, approximately 1 in every 600 women attending breast-screening programmes in the United Kingdom is diagnosed with breast carcinoma *in situ* (BCIS). This amounted to almost 3000 cases in 2004–2005 (NHS Cancer Screening Programmes, 2006). The most common form of treatment is lumpectomy, with or without radiotherapy, although women with a more diffuse pattern of BCIS may undergo mastectomy. A number of previous studies have focused on the risk of second breast cancer following a diagnosis of BCIS (Habel *et al*, 1997; Wärnberg *et al*, 2000; Claus *et al*, 2003; Levi *et al*, 2005; Rawal *et al*, 2005), with estimates of relative risk generally of the order of two- to fivefold. Few studies have looked at the occurrence of subsequent invasive cancers at other sites (Ward *et al*, 1992; Franceschi *et al*, 1998; Soerjomataram *et al*, 2006), and these were based on relatively small numbers of cases.

In the current population-based study, we have investigated the occurrence of subsequent invasive cancers in a very large series of unselected women with BCIS in southeast England. We have also examined the relationship between treatment for BCIS and the development of subsequent cancers.

## Materials and methods

All recorded cases of BCIS (ICD10 code D05) diagnosed in women between 1971 and 2003 were extracted from the Thames Cancer Registry (TCR) database. The TCR is a population-based registry covering London and a large part of southeast England. Patients with any other cancers diagnosed prior to, or at the same time as, BCIS were excluded in order to avoid confounding the sequelae of BCIS with those of other cancers, particularly breast cancer. The occurrence of subsequent cancers was analysed by calculating the standardised incidence ratio (SIR) for each cancer site. This is the ratio of the observed number of cancers at that site divided by the number expected in the general population, calculated by multiplying time at risk by age/calendar period-matched incidence rates for women in southeast England. For each case, person-years were calculated from date of diagnosis to the end of 2004, date of death, date of last known follow-up or date of diagnosis of subsequent cancer, whichever was earlier. Ipsilateral and contralateral subsequent breast cancers were considered separately. When analysing ipsilateral subsequent breast cancer, follow-up was truncated at the date of mastectomy if this had been performed.

Information on radiotherapy and hormonal therapy received within 6 months of diagnosis was also available on the database. Although precise details of the type of hormonal therapy were unavailable, the use of tamoxifen is likely to predominate. The effects of treatment on the occurrence of subsequent cancers were explored by applying Cox regression models with age at diagnosis of BCIS (stratified into 5-year age groups), radiotherapy and hormonal therapy as covariates. Results for a given mode of treatment were expressed as the hazard ratio (HR) in those with a record of receiving that treatment *vs* those with no such record, adjusted for age and the receipt or otherwise of the other treatment mode.

## Results

A total of 14 329 cases of BCIS were extracted from the database. Of these, 23 were excluded as having a recorded date of diagnosis that was the same as the date of death, and a further 1046 cases were excluded as having a prior or synchronous cancer. An additional 424 patients who were recorded as receiving chemotherapy were also excluded, as it is likely that these patients were wrongly classified as chemotherapy was not a normal component of treatment for BCIS during the period of the study. This left a total of 12 836 cases for analysis. Table 1 shows the characteristics of the patients included in the study. The majority (86%) of the *in situ* cancers were intraductal BCIS, 5% were lobular BCIS and the remainder were of other or mixed morphological subtypes. The mean age at diagnosis was 57 years. Overall, 3880 patients (30.2%) had a record of radiotherapy treatment and 3064 (23.9%) had a record of hormonal therapy. Of the patients who underwent breast-conserving surgery, 42% had a record of treatment with radiotherapy.

Table 2 shows the observed and expected numbers of subsequent invasive cancers at a number of major sites. A greater than expected number of subsequent occurrences of cancer was found for two sites: breast (C50) and corpus uteri (C54). Standardised incidence ratios were 1.96 for breast cancer (95% confidence interval (CI) 1.79–2.14) and 1.42 for uterine cancer (95% CI 1.11–1.78). There was a reduced incidence of cancers of the pancreas (SIR 0.59; 95% CI 0.37–0.90) and lung (SIR 0.75; 95% CI 0.61–0.92), and of cancers at ‘other’ sites (SIR 0.52; 95% CI 0.41–0.66).

Table 3 shows the SIRs for these two sites by time since diagnosis of BCIS, and also separately for subsequent ipsilateral and contralateral breast cancers. The overall SIRs for subsequent ipsilateral and contralateral breast cancers were 2.37 (95% CI 2.07–2.70) and 1.72 (95% CI 1.53–1.93), respectively. For subsequent ipsilateral breast cancer, the excess was fairly constant, with SIRs of 2.24, 2.55 and 2.27 at times 0–1, 1–5 and >5 years after BCIS diagnosis, respectively. For subsequent contralateral breast cancer, there was a steady decline in the excess over time, with SIRs of 3.00, 2.07 and 1.38, respectively, in these three time periods. For subsequent cancer of the uterus, the excess became statistically significant only at >5 years after BCIS diagnosis (SIR 1.58; 95% CI 1.18–2.07).

In general, results were similar when looking separately at patients diagnosed with ductal carcinoma *in situ* (DCIS) or lobular carcinoma *in situ* (LCIS) (results not shown). However, in the first year post-diagnosis, there was a significant excess of subsequent ipsilateral breast cancers in women diagnosed with LCIS (SIR 8.06; 95% CI 2.62–18.82), but not for DCIS (SIR 1.51; 95% CI 0.80–2.58).

The results of the Cox regression analysis are shown in Table 4. The risk of subsequent uterine cancer was significantly increased in women with a record of hormonal therapy (compared with those without), with an HR of 2.97 (95% CI 1.84–4.80). The risk of subsequent breast cancer was decreased in women with a record of radiotherapy (HR 0.73; 95% CI 0.60–0.89); this reduction in risk was due solely to a decreased risk of subsequent ipsilateral breast cancer (HR 0.40; 95% CI 0.29–0.55). There was no significant reduction in the risk of subsequent contralateral breast cancer in women with a record of radiotherapy. Including period of diagnosis and morphological type as additional factors in the model had no significant effect on the estimated HRs.

A separate analysis restricted to women in the age range of 50–64 years eligible for breast screening showed essentially the same results (data not shown). In addition, stratifying the analysis into pre- and post-screening periods (1971–1989 *vs* 1990–2003) produced similar results with respect to subsequent invasive breast cancer. There was no significant excess of uterine cancer in the earlier period, but this was due to the small number of patients receiving hormonal therapy prior to mid 1980s/early 1990s.

## Discussion

We have found an excess of subsequent cancers of the breast and corpus uteri following a diagnosis of BCIS. For subsequent uterine cancer, the excess became statistically significant only at >5 years after BCIS diagnosis, consistent with a treatment effect. This was confirmed by Cox regression analysis: the risk of subsequent uterine cancer was significantly increased in women with a record of hormonal therapy, with an HR of 2.97 (95% CI 1.84–4.80).

Increased occurrence of breast cancer following BCIS has been shown in a number of previous studies from around the world: from Sweden (Wärnberg *et al*, 2000; Rawal *et al*, 2005); Switzerland (Franceschi *et al*, 1998; Levi *et al*, 2005); The Netherlands (Soerjomataram *et al*, 2006); and the United State of America (Ward *et al*, 1992; Habel *et al*, 1997; Claus *et al*, 2003). Soerjomataram *et al* (2006) also found an increased occurrence of skin cancer (SIR 1.7; 95% CI 1.1–2.6) following BCIS, whereas Ward *et al* (1992) found that (apart from breast cancer) colorectal, cervical and endometrial cancers were the most prevalent in BCIS patients. On the other hand, Franceschi *et al* (1998) found no excess risk at any cancer site other than the breast.

Soerjomataram *et al* (2006) studied subsequent ipsilateral and contralateral breast cancers separately, and found the increased risks to be similar (SIR for ipsilateral 1.9; contralateral 2.0) – although each of these figures was based on a per-person rather than per-breast calculation and should therefore be doubled in order to be directly comparable with our SIR estimates.

The very high SIR (3.00; 95% CI 2.17–4.04) observed for subsequent contralateral breast cancer in the first year after BCIS diagnosis may be a manifestation of co-existing disease, or may result from the extra surveillance around the time of treatment for the first breast cancer, intense medical follow-up or self-observation of the patient – resulting in either detection or lead-time bias. The fact that detection bias is in operation is further indicated by the high risk of ipsilateral cancer (SIR 8.06) in patients with LCIS during the first year of follow-up, as LCIS has traditionally been thought to be a marker for high risk of multifocal and contralateral disease (Frykberg *et al*, 1987; Page *et al*, 1991; Chuba *et al*, 2005).

The SIR values we found for subsequent breast and uterine cancers are similar to those following invasive breast cancer from an earlier study on the same population (Evans *et al*, 2001). This similarity was also seen in the studies by Soerjomataram *et al* (2006) and Claus *et al* (2003).

It is known from randomised controlled trials that radiotherapy after breast-conserving surgery for BCIS reduces recurrences in the ipsilateral breast (Fisher *et al*, 1993; Fisher *et al*, 1998; Julien *et al*, 2000; Houghton *et al*, 2003; Bijker *et al*, 2006; Emdin *et al*, 2006; Ringberg *et al*, 2007). We also found this protective effect of radiotherapy on the subsequent development of invasive cancer in the ipsilateral breast (HR 0.40; 95% CI 0.29–0.55). This HR is similar to those reported from the clinical trials. Thus, the well-studied effects of radiotherapy appear to hold true in an observational setting. However, our findings are in contrast with those of Soerjomataram *et al* (2006), who found (also in an observational setting) that both ipsilateral and contralateral invasive breast cancer risks were slightly higher in BCIS patients who received radiotherapy.

In our study, we found a significant effect of hormonal therapy on the subsequent risk of uterine cancer in women diagnosed with BCIS, with those who received this form of treatment being three times more likely to develop cancer of the uterus than those who did not. This was not offset by any significant reduction in the risk of developing subsequent invasive breast cancer (HR 1.01; 95% CI 0.82–1.25).

A number of previous studies have demonstrated a link between hormonal treatment for invasive breast cancer and the development of uterine or endometrial cancer (Khandekar *et al*, 1978; Hardell, 1988; Fornander *et al*, 1989; Atlante *et al*, 1990; Gusberg, 1990; Mathew *et al*, 1990; Andersson *et al*, 1991; Fisher *et al*, 1994; Rubino *et al*, 2003). However, it is generally accepted that for women with invasive breast cancer, the benefits of hormonal treatment, such as tamoxifen, outweigh the associated risks (Gail *et al*, 1999).

In the NSABP B-24 study, Fisher *et al* (1999) compared lumpectomy plus radiation therapy with lumpectomy, radiation therapy and tamoxifen treatment. They found that the risk of ipsilateral breast cancer was lower in the tamoxifen group. In contrast, Houghton *et al* (2003) in the UK randomised trial found that ipsilateral invasive disease was not reduced by tamoxifen, and concluded that ‘there is little evidence for the use of tamoxifen in these women’.

We found significantly lower than expected numbers of lung and pancreatic cancers following a diagnosis of BCIS as well as in the group of ‘other’ cancers. This has not been reported in other studies. The incidence of breast cancer and the uptake of mammographic screening are known to be related to the socioeconomic status, and it is likely that the women in our study are more affluent and generally more health conscious, with lower than average rates of smoking.

The major strengths of our study are its population-based design, the large number of patients and the length of follow-up. However, it also has a number of limitations. The findings on the incidence of second primary cancers are based on cancer registry data, which contain limited and incomplete treatment information largely confined to initial management and lacking in detail of specific drugs, radiation doses and radiation fields. Also, there may be underreporting of second cancers in those who leave the registry catchment area. In the majority of such cases, the registry would neither be informed of the patient's emigration nor receive notification of subsequent cancers. This would lead to an underestimation of the risk of subsequent cancer, and hence the quoted SIR values relating to excess risk will tend to be erroneous on the conservative side. In addition, as the data were extracted in 2005, cancer incidence till the end of 2004 would still be incomplete by a few percent. Again, this would tend to lead to an underestimation of SIRs, although this problem would be restricted to the short-term estimates.

There is also a possibility of under-ascertainment of treatment in the TCR database, as information on treatment more than 6 months after diagnosis is not recorded. This is more likely to be of importance in relation to the recording of radiotherapy, as waiting times for radiotherapy are known to have increased in recent years (Robinson *et al*, 2005; Jack *et al*, 2007). However, any misclassification would result in a dilution of the difference between the treated and untreated groups, and hence any reported estimates of treatment effects would be conservative. A previous study on the same database (Roychoudhuri *et al*, 2004), looking at malignancies following radiotherapy for invasive breast cancer, showed an increase in myeloid leukaemia consistent with other studies, suggesting that the treatment data are reliable.

The quality and completeness of the recording of BCIS have been variable over the period studied. There was an increase in the numbers in mid 1990s, corresponding to an increased rate of detection following the introduction of the national breast-screening programme. Since then, rates have continued to rise, with the overall age-standardised rate for 2003 being approximately 13 per 100 000 women. Our identification of BCIS cases was based on patient pathology reports. As a result of changes over time in the histological classification and diagnosis of BCIS, it is possible that some of our patients may have had invasive breast cancer that was missed on diagnosis, but we would expect the number of such cases to be small.

There have also been significant changes in treatment for BCIS over the period of the study. Cox regression analysis indicates that after allowing for these changes in treatment over time, the period of diagnosis was not independently related to subsequent risk of cancer, that is, there is no significant effect of period over and above the associated treatment effects.

Although we have allowed for the effects of curative mastectomies by analysing ipsilateral and contralateral subsequent breast cancers separately, our data set may still contain women who underwent prophylactic removal of the contralateral breast, which we were unable to allow for. However, the number of such women is likely to be small and would have minimal effect on the calculation of risk estimates.

In many women, BCIS is detected at screening. Our data show that such women are at a moderately increased risk of a subsequent contralateral invasive breast cancer, a greater risk of ipsilateral invasive breast cancer in those with a preserved breast (especially if radiotherapy is not given) and a risk of uterine cancer associated with hormonal treatment. However, all three can be managed – the first by surveillance to detect and treat early, the second by radiotherapy and the third by the limitation of the administration of tamoxifen to those patients for whom it is really indicated.

## Figures and Tables

**Table 1:** Patient characteristics

	**No. of patients**	**% of patients**
*Age at diagnosis (years)*		
<45	1764	13.7
45–49	1611	12.6
50–54	2568	20.0
55–59	2133	16.6
60–64	1920	15.0
⩾65	2840	22.1
		
*Period of diagnosis*		
1971–1974	971	7.6
1975–1984	2360	18.4
1985–1994	3892	30.3
1995–2003	5613	43.7
		
*Morphological type*		
Ductal	11 052	86.1
Lobular	681	5.3
Other/mixed	1103	8.6
		
*Surgery*		
Total mastectomy	4713	36.7
Partial mastectomy	764	6.0
Lumpectomy	4991	38.9
Other surgery	632	4.9
None	1736	13.5
		
*Radiotherapy*		
Yes	3880	30.2
No	8956	69.8
		
*Hormonal therapy*		
Yes	3064	23.9
No	9772	76.1
Total cases	12 836	

**Table 2:** Occurrence of invasive cancers at specific sites subsequent to breast carcinoma *in situ*

**Site**	**Observed**	**Expected**	**SIR**	**95% CI**
Head and neck	19	25.03	0.76	0.46–1.19
Oesophagus	22	22.85	0.97	0.60–1.46
Stomach	26	32.82	0.79	0.52–1.16
Colorectal	130	147.93	0.88	0.73–1.04
Pancreas	22	37.20	0.59	0.37–0.90
Lung	101	133.92	0.75	0.61–0.92
Malignant melanoma	14	21.80	0.64	0.35–1.08
Breast	512	261.44	1.96	1.79–2.14
Cervix uteri	12	20.39	0.59	0.30–1.03
Corpus uteri	74	52.14	1.42	1.11–1.78
Ovary	49	59.81	0.82	0.61–1.08
Kidney	16	16.02	1.00	0.57–1.62
Bladder	22	30.13	0.73	0.46–1.11
Non-Hodgkin's lymphoma	24	35.56	0.67	0.43–1.00
Multiple myeloma	11	16.00	0.69	0.34–1.23
Leukaemia	20	24.93	0.80	0.49–1.24
Others^a^	72	138.28	0.52	0.41–0.66
All sites^a^	1146	1076.25	1.06	1.00–1.13

CI=confidence interval; SIR=standardised incidence ratio.

a Excluding non-melanoma skin cancers.

**Table 3:** Occurrence of invasive cancers of the breast and uterus subsequent to breast carcinoma *in situ* (by time since diagnosis)

	**Years since diagnosis**			
**Site**	**0–1**	**1–5**	**>5**	**Total**
*Uterus*				
Pyrs	12 736	42 110	73 850	128 696
Obs	6	16	52	74
Exp	4.11	15.07	32.96	52.14
SIR	1.46	1.06	1.58	1.42
CI	0.54–3.18	0.61–1.72	1.18–2.07	1.11–1.78)
				
*Breast*				
*All*				
Pyrs	10 467	33 671	53 206	97 344
Obs	66	189	257	512
Exp	24.57	83.59	153.28	261.44
SIR	2.69	2.26	1.68	1.96
CI	2.08–3.42	1.95–2.61	1.48–1.89	1.79–2.14
				
*Ipsilateral*				
Pyrs	8388	26 106	35 250	69 744
Obs	23	86	117	226
Exp	10.25	33.73	51.47	95.45
SIR	2.24	2.55	2.27	2.37
CI	1.42–3.37	2.04–3.15	1.88–2.72	2.07–2.70
				
*Contralateral*				
Pyrs	12 547	41 235	71 162	124 944
Obs	43	103	140	286
Exp	14.32	49.86	101.81	165.99
SIR	3.00	2.07	1.38	1.72
CI	2.17–4.04	1.69–2.51	1.16–1.62	1.53–1.93

CI=confidence interval; Exp=expected number; Obs=observed number; Pyrs=person-years; SIR=standardised incidence ratio.

**Table 4:** HRs and 95% CIs for subsequent cancer in relation to type of treatment

	**Type of treatment**	
**Site**	**Radiotherapy**	**Hormone therapy**
*Uterus*		
Pyrs^a^	41 554	26 866
*n*^b^	22	34
HR^c^	0.83	2.97
CI	0.50–1.37	1.84–4.80
		
*Breast*		
*All*		
Pyrs^a^	34 105	22 834
*n*^b^	145	120
HR^c^	0.73	1.01
CI	0.60–0.89	0.82–1.25
		
*Ipsilateral*		
Pyrs^a^	27 803	19 387
*n*^b^	49	65
HR^c^	0.40	1.20
CI	0.29–0.55	0.89–1.61
		
*Contralateral*		
Pyrs^a^	40 408	26 281
*n*^b^	96	55
HR^c^	1.07	0.85
CI	0.84–1.37	0.63–1.15

CI=confidence interval; HR=hazard ratio; Pyrs=person-years.

a Total person-years in group receiving treatment of specified type.

b Number of occurrences of subsequent cancer in group receiving treatment.

c HR for treatment recorded *vs* not recorded, adjusted for 5-year age group and other treatment mode.
